# Optimization and Technical Considerations for the Dye-Exclusion Protocol Used to Assess Blood–Brain Barrier Integrity in Adult *Drosophila melanogaster*

**DOI:** 10.3390/ijms24031886

**Published:** 2023-01-18

**Authors:** Kesshni Bhasiin, Olivia Heintz, Kenneth J. Colodner

**Affiliations:** Program in Neuroscience and Behavior, Mount Holyoke College, South Hadley, MA 01075, USA

**Keywords:** *Drosophila melanogaster*, blood–brain barrier, traumatic injury, dye-exclusion assay, barrier integrity

## Abstract

The blood–brain barrier (BBB) is a multicellular construct that regulates the diffusion and transport of metabolites, ions, toxins, and inflammatory mediators into and out of the central nervous system (CNS). Its integrity is essential for proper brain physiology, and its breakdown has been shown to contribute to neurological dysfunction. The BBB in vertebrates exists primarily through the coordination between endothelial cells, pericytes, and astrocytes, while invertebrates, which lack a vascularized circulatory system, typically have a barrier composed of glial cells that separate the CNS from humoral fluids. Notably, the invertebrate barrier is molecularly and functionally analogous to the vertebrate BBB, and the fruit fly, *Drosophila melanogaster*, is increasingly recognized as a useful model system in which to investigate barrier function. The most widely used technique to assess barrier function in the fly is the dye-exclusion assay, which involves monitoring the infiltration of a fluorescent-coupled dextran into the brain. In this study, we explore analytical and technical considerations of this procedure that yield a more reliable assessment of barrier function, and we validate our findings using a traumatic injury model. Together, we have identified parameters that optimize the dye-exclusion assay and provide an alternative framework for future studies examining barrier function in *Drosophila*.

## 1. Introduction

The blood–brain barrier (BBB) is a multicellular construct that creates a restrictive and protective barrier between the CNS and the rest of the body. The BBB regulates the diffusion and transport of ions, metabolites, and inflammatory mediators, and bars the entry of unwanted blood-borne factors into the brain [1]. BBB disruption can disrupt homeostasis and damage brain cells, and BBB abnormalities are associated with a wide range of neurodegenerative disorders [2,3,4].

While the extent to which BBB disruption contributes to neurodegenerative disease pathogenesis remains to be defined, animal models are valuable systems in which to interrogate the cellular and molecular mechanisms that regulate BBB integrity. Both vertebrate and invertebrate model systems have proven useful in studying BBB function despite their different circulatory systems. Vertebrates possess a closed circulatory system with blood flow restricted to blood vessels, while invertebrates, such as *Drosophila melanogaster*, possess an avascular, open circulatory system in which hemolymph is pumped throughout the body [5]. These fundamental differences notwithstanding, the *Drosophila* hemolymph-brain barrier (henceforth referred to as BBB for simplicity of comparison) restricts and protects the brain from the periphery and is functionally and molecularly similar to the vertebrate BBB [6,7]. Both barriers are composed of tight/septate junctions, and glial cells play a prominent role in regulating barrier integrity [1,8]. As such, *Drosophila* is increasingly recognized as a robust model system to investigate barrier function, particularly given the array of genetic tools available to fly researchers.

The existence of a mechanical barrier between the CNS and periphery was proposed when it was observed that intravenously administered dyes and pharmacological substances were typically excluded from the brain and spinal cord [9]. Dye exclusion was later shown to be mediated by tight junctions between neighboring endothelial cells [10], and this dye-exclusion technique has proven to be a useful paradigm to assess BBB integrity. A similar technique has been developed for *Drosophila* where the injection of fluorescent-coupled tracers into the body cavity is monitored for exclusion from the CNS as a measure of brain-barrier integrity [11,12]. This technique has been used to identify key structural proteins and signaling pathways that regulate BBB development and function in *Drosophila* [13,14,15], and has also been used to demonstrate that traumatic injury and glial dysfunction can impact BBB integrity [16,17]. However, the appropriate accumulation of fluorescence at the glial cell barrier, which is itself an indicator of proper barrier integrity, confounds the researcher’s effort to quantify fluorescence that has infiltrated the brain.

In this study, we have performed a detailed analysis of fixation and imaging conditions related to the dye-exclusion assay, and we have identified parameters that improve reliability and reduce technical variability for the procedure. We also demonstrate the utility of these modifications using a traumatic injury paradigm. Together, these findings provide an optimized workflow to assess barrier integrity in *Drosophila*, and should prove useful for researchers interrogating barrier function.

## 2. Results

### 2.1. Intraparenchymal Binary Analysis More Accurately Reflects Dye Infiltration Than Single-Channel Region of Interest (ROI) Analysis

**Figure 1:**
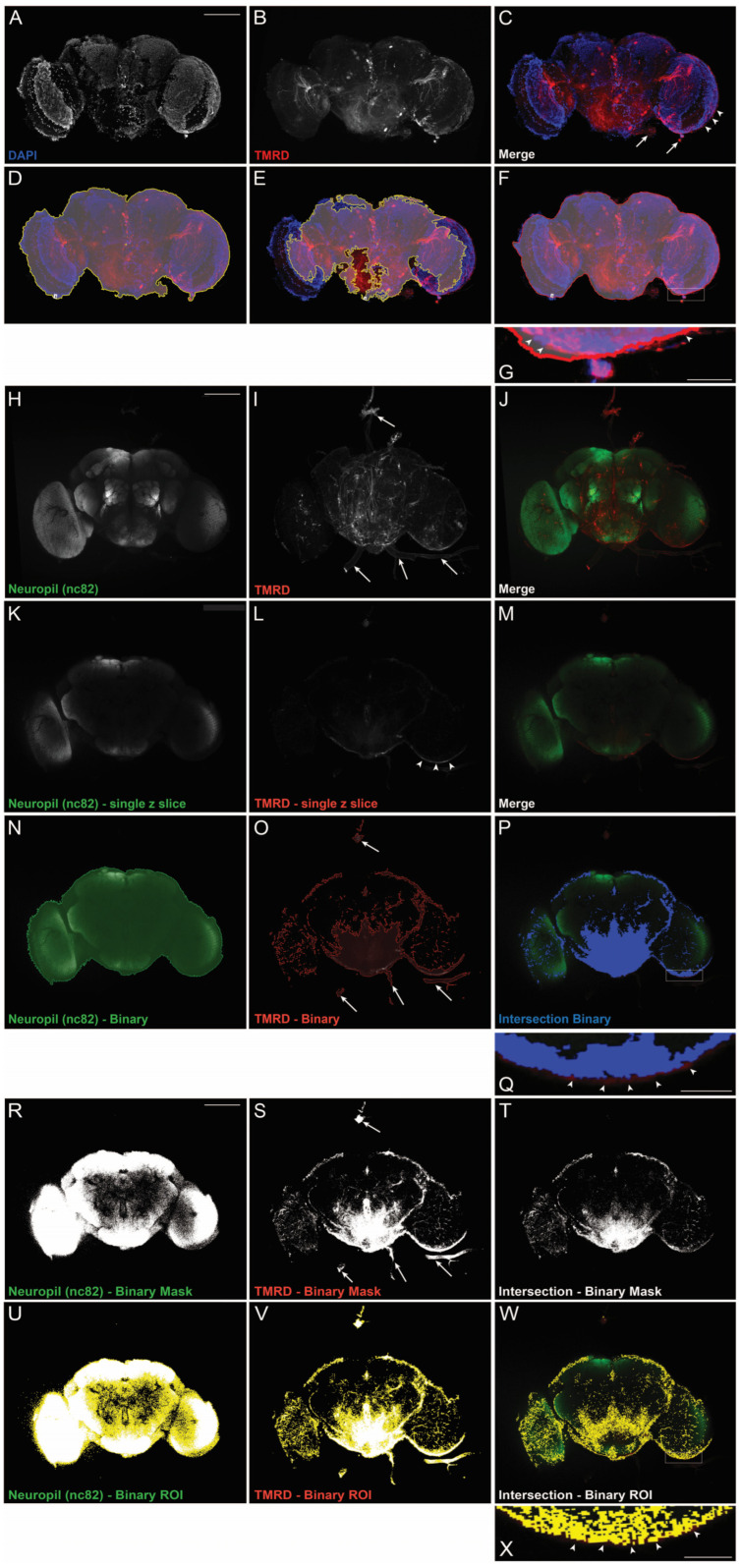
Different methods of assessing dye infiltration into the adult *Drosophila* brain. (**A**–**G**) Representative maximum projection laser-scanning confocal images of a d10 *w^1118^* female whole-mount brain injected with 10 kDa tetramethylrhodamine dextran (TMRD) on d9 showing: (**A**) DAPI, (**B**) TMRD, and (**C**) merged channels. White arrows point to structures outside the brain that have taken up TMRD, and white arrowheads point to TMRD at the glial barrier. (**D**–**G**) NIS Elements software autodetection of (**D**) an overestimated ROI (shaded region), (**E**) an underestimated ROI (shaded region), and (**F**) manual correction of ROI to exclude structures outside the brain (arrows in (**C**)). Inset in (**G**) shows TMRD at the glial barrier (arrowheads) that is arbitrarily included in the ROI. (**H**–**J**) Representative maximum projection laser-scanning confocal images of a d10 *w^1118^* female whole-mount brain injected with TMRD on d9 showing: (**H**) Neuropil (nc82) staining, (**I**) TMRD, and (**J**) merged channels. (**K**,**L**) Single z-plane from the same brain in (**H**–**J**) showing: (**K**) Neuropil (nc82) staining, (**L**) TMRD, and (**M**) merged channels. Arrowheads in (**L**) point to TMRD at the glial barrier. (**N**–**Q**) NIS Elements binary analysis of a single z-slice image showing: (**N**) Neuropil (nc82) binary alone, (**O**) TMRD binary alone, and (**P**) nc82/TMRD intersection binary. Arrows in (**O**) show TMRD staining outside the brain that is included in the binary (**O**), but excluded from the intersection binary (**P**). Inset in (**Q**) shows TMRD at the blood–brain barrier (arrowheads) that is not included in the intersection binary. (**R**–**T**) FIJI binary analysis of the same z-slice in (**H**–**J**) showing: (**R**) Neuropil (nc82) binary mask alone, (**S**) TMRD binary mask alone, and (**T**) nc82/TMRD intersection binary. Arrows in (**S**) show TMRD staining outside the brain that is included in the binary mask (**S**), but excluded from the intersection binary mask (**T**). (**U**–**X**) FIJI autodetection of ROIs on (**U**) Neuropil (nc82) staining, (**V**) TMRD, and (**W**) nc82/TMRD intersection binary masks. Inset in (**X**) shows the intersection ROI overlaid over fluorescence at the glial barrier (arrowheads). Scale bar in (**A**–**F**); (**H**–**P**); (**R**–**W**) = 100 µm. Scale bar in insets in (**G**,**Q**,**X**) = 20 µm.

The dye-exclusion assay is routinely used to monitor BBB integrity to further our understanding of the barrier between the nervous system and the periphery. In *Drosophila*, this assay is performed by injecting a fluorescent-coupled dextran (10 kDa tetramethylrhodamine dextran (TMRD)) into the body of the fly, and monitoring its exclusion from the brain by measuring the amount of fluorescence present in a dissected whole-mount brain [12]. We injected TMRD into 9-day-old *w^1118^* flies, dissected the brain the following day, and then used laser-scanning confocal microscopy to image and quantify the fluorescence that infiltrated the brain, comparing two methods of analysis. For the region of interest (ROI) analysis, we generated maximum projection images from acquired z-stacks for the DAPI and TMRD channels and used the DAPI channel to define the brain volume (Figure 1A–C). An ROI is drawn around the brain, and subsequent measurement of mean TMRD-derived fluorescence intensity is quantified from these ROIs to determine the amount of tracer that has entered the brain. To allow for unbiased ROI generation, we used NIS Elements software to automatically determine the ROI that circumscribed DAPI positive cells and found that the automation of the process lends itself to both overestimation (Figure 1D) and underestimation (Figure 1E) of the brain area. Furthermore, the trachea, fat body, and other external structures that were positive for TMRD around the brain (arrows in Figure 1C) were often included in the ROI (Figure 1D). Manually correcting for these errors is possible, but is subjective and could also lead to the exclusion of fluorescence that is actually within the brain (Figure 1F). Moreover, researchers must decide how to consider the fluorescence that is retained within the glial barrier surrounding the brain, which itself is a reflection of proper barrier integrity and dye exclusion (Figure 1G). Additionally, given that a maximum projection image is a two-dimensional representation of a three-dimensional brain, each pixel only takes into account the brightest pixels at a specific x–y coordinate and does not include dimmer pixels at a different z depth of that same x–y coordinate. Taken together, the ROI analysis of the dye-exclusion assay has notable limitations.

We developed an alternative analysis paradigm that better accounts for the three-dimensional brain volume, and allows for the impartial inclusion/exclusion of TMRD-related fluorescence. This binary approach includes immunostaining for the neuropil-specific protein, nc82, in conjunction with TMRD imaging (Figure 1H–M). Laser-scanning confocal z-stack volumes are acquired, and nc82 and TMRD channels are separately thresholded to define a binary for each channel at each individual z-slice (Figure 1N,O; Supplementary Figure S1). These two binaries are overlaid and analyzed for overlap to determine an intersection binary (Figure 1P). This process ensures that any tracer-derived fluorescence is measured only from regions within the brain parenchyma, and therefore only captures TMRD that has infiltrated the brain. Accessory brain tissue, such as the trachea and fat body, is automatically excluded from this analysis, in addition to fluorescence that has been retained within the glial barrier itself (Figure 1P,Q). 

Given that this initial approach utilized a specialized Nikon NIS Elements software, we considered whether open-source software, such as FIJI/ImageJ, could also be used to carry out a similar analysis. We found that the nc82 and TMRD channels could be individually thresholded in FIJI and that an intersection binary mask of these two channels could be generated (Figure 1R–T). Regions of interest were automatically created across each z-slice of the intersection binary mask and these regions were utilized to measure tracer-derived fluorescence (Figure 1U–W). Notably, while TMRD-positive accessory brain tissue was excluded from the intersection of the two binary masks (arrows in Figure 1S compared to Figure 1T), we observed only partial exclusion of the glial barrier using FIJI analysis conditions (Figure 1W,X). However, for both FIJI and Nikon software methodologies, we found that including thresholded layers at each optical z-slice within the brain volume, allows one to obtain a three-dimensional intersection binary that quantifies TMRD-derived fluorescence for the entire brain volume.

### 2.2. Whole-Body Pre-Fixation Reduces Variability of Dye-Infiltration Assay in Adult Drosophila

**Figure 2:**
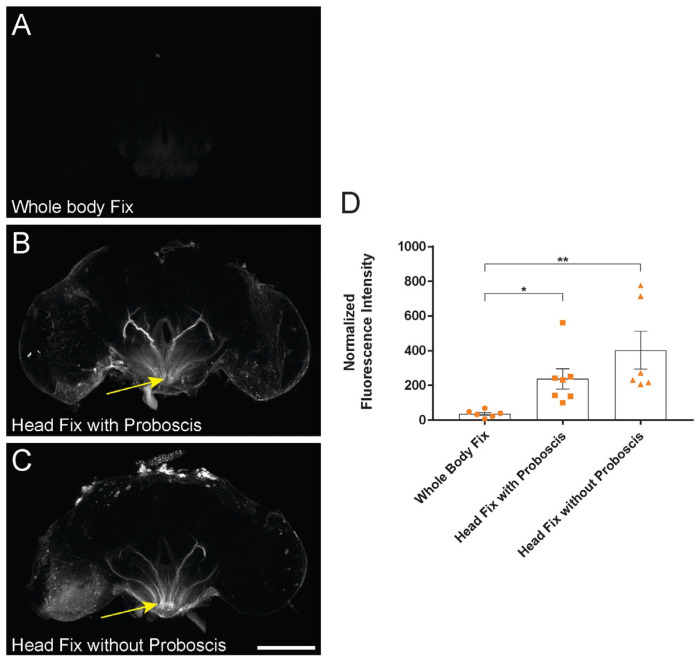
Whole-body pre-fixation reduces baseline dye infiltration. (**A**–**C**) Representative maximum projection laser-scanning confocal images of whole-mount brains from d10 *w^1118^* females injected with 10 kDa TMRD on d9. Baseline TMRD infiltration in: (**A**) whole-body pre-fixation, (**B**) head pre-fixation with proboscis attached, and (**C**) head pre-fixation without proboscis. TMRD infiltration into the subesophageal zone (SEZ) was noted in both head pre-fixation conditions (arrows in (**B**,**C**)). (**D**) Quantification of normalized TMRD fluorescence infiltration across fixation conditions using nc82/TMRD intersection binary analysis. * *p* < 0.05; ** *p* < 0.01; Kruskal–Wallis test with Dunn’s multiple comparisons; n = 6–7; error bars are presented as mean ± SEM. Scale bar = 100 µm. Maximum projection images were generated from the middle ~75 µm region of the brain to highlight SEZ infiltration.

To further optimize the dye-infiltration technique, we compared three fixation strategies to determine if the baseline fluorescence of the assay is impacted by pre-imaging specimen processing. We injected 9-day-old *w^1118^* flies and subjected them to three fixation conditions: whole-body pre-fixation and head pre-fixation with or without the proboscis removed. We found that baseline fluorescence was significantly reduced when flies underwent whole-body fixation before the brains were dissected and imaged (Figure 2). Conditions where the head was removed prior to fixation, both with and without proboscis removal, were more variable and yielded higher baseline TMRD infiltration and fluorescence. This increased TMRD infiltration was often associated with elevated fluorescence localized to the subesophageal zone (SEZ) (Figure 2B,C), and may indicate that the mechanical force of removing the fly’s head or proboscis during dissection can physically disrupt the BBB. The whole-body pre-fixation, therefore, provides an analysis strategy where the TMRD found within the brain is more indicative of the experimental variables under examination, and not related to how the brain is processed during the dissection. 

### 2.3. Whole-Body Fixation Better Reveals the Effect of Traumatic Injury on Barrier Integrity

**Figure 3:**
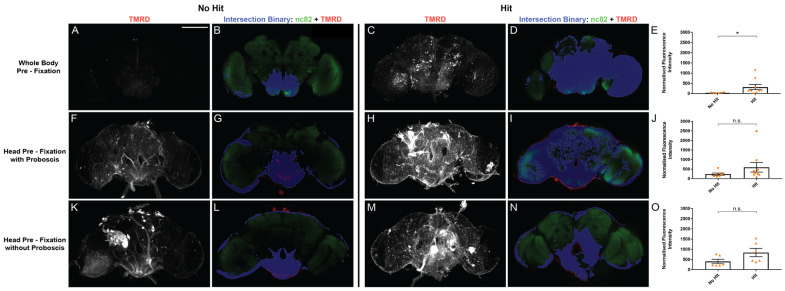
Whole-body pre-fixation reduces baseline TMRD fluorescence and reveals the effect of traumatic injury on dye infiltration. (**A**,**C**,**F**,**H**,**K**,**M**) Representative maximum projection laser-scanning confocal images of the TMRD channel and (**B**,**D**,**G**,**I**,**L**,**N**) single z-plane intersection binary of nc82 and TMRD signal, of whole-mount brains from d10 *w^1118^* females injected with TMRD on d9. Brains were processed for fixation by: (**A**–**D**) whole-body pre-fixation, (**F**–**I**) head pre-fixation with proboscis attached, (**K**–**N**) head pre-fixation without proboscis. Flies either did not experience a traumatic injury (No Hit) or were exposed to traumatic injury 2 h after dye injection (Hit). (**E**,**J**,**O**) Quantification of normalized TMRD fluorescence infiltration across fixation conditions using nc82/TMRD intersection binary analysis. * *p* < 0.05; n.s. = not significant. Mann–Whitney U-test (**E**,**J**), *t*-test (**O**); n = 6–9. Error bars are presented as mean ± SEM. Scale bar = 100 µm.

To determine if our alternative strategy to the dye-infiltration assay yields biologically relevant results, we assessed whether fixation conditions could affect known external factors that influence barrier integrity. Traumatic injury has been shown to induce barrier disruption in the adult fly, and the dye-exclusion assay is often used to identify factors that influence this injury-induced disruption [16,17]. To determine if pre-fixation conditions affected tracer infiltration, we subjected 9-day-old *w^1118^* flies to traumatic injury and assessed whether the method of fixation influenced injury-associated barrier disruption. The traumatic injury was induced via the spring-loaded high-impact trauma device routinely used to inflict full-body injury to adult *Drosophila* [16,18]. We found that TMRD infiltration, as a read-out of barrier disruption, was elevated in flies that experienced a traumatic injury, but only the whole-body pre-fixation condition yielded a statistically significant increase in response to injury (Figure 3). Fixation conditions involving head removal (with or without the proboscis) both showed a trend toward an injury-induced increase in TMRD infiltration, but the elevated baseline fluorescence of the no-hit conditions may have masked the impact of injury on the barrier disruption. Taken together, these data support that whole-body fixation, before the brain is dissected, reduces background baseline fluorescence of the dye-infiltration assay, and allows for better assessment of the effects of traumatic injury on CNS barrier integrity in adult *Drosophila*. 

## 3. Discussion

In this study, we provide an alternative strategy for performing the dye-infiltration assay that is routinely used to assess barrier integrity in adult *Drosophila*. We found that a parenchyma-specific, three-dimensional approach to analysis, in conjunction with whole-body pre-fixation, provides researchers with a comprehensive and unbiased methodology toward examining CNS barrier function. 

Nervous system barriers are critical for homeostatic regulation and their dysfunction is increasingly recognized as a pathological event in the onset and progression of neurodegenerative disorders [1,7]. *Drosophila*, with its short lifespan and array of genetic tools, is an effective model system to identify factors that contribute to barrier function and integrity. The dye-exclusion assay, which takes advantage of the fact that fluorescent-coupled dextrans (e.g., TMRD) are normally excluded from the fly brain, is a useful technique in monitoring barrier function. To perform this assay, researchers quantify dye fluorescence within the brain and use this as a measure of barrier disruption. Typically, dye fluorescence is measured within individual planes of an optical section through the middle of the brain or through an ROI around a maximum intensity projection of the entire brain to assess dye infiltration. This technique has yielded important findings about the molecular mechanisms that control barrier function, and the consequences of its disruption [14,16,17], but determining the boundaries of the ROI, or the specific plane of the section to use for analysis, can be subjective. Moreover, whether or not to include the dye that is trapped within the glial barrier along the outside of the fly brain (an indicator that the barrier is appropriately excluding the dye) is not explicitly defined by the protocol. The analysis we describe herein builds upon the established dye-exclusion assay and provides an unbiased method that restricts the analysis of infiltrated fluorescent dye within the internal brain parenchyma. Bruchpilot, a pre-synaptic protein enriched in *Drosophila* neuropil, is recognized with the antibody nc82 and is routinely used as a counterstain for immunofluorescence experiments [19]. By creating two thresholded binaries: one of the nc82 stain, and the other of the fluorescent dye, and determining their intersection, this new methodology ensures that all fluorescent dye included in the analysis is localized within the brain parenchyma and, therefore, represents true infiltration into the brain (Figure 1). Importantly, nc82 staining is compartmentalized internally within the fly brain and is not found at the glial barrier. This ensures that dye that may be trapped within the glial barrier, or found in other structures outside the brain, will be automatically excluded from analysis, as they will lack nc82 staining. One caveat to note is that dye that has infiltrated the brain but resides within the brain cortex that typically lacks nc82 staining, will also be excluded from analysis. This intersection binary approach, therefore, calculates parenchyma-specific infiltrated dye only. Furthermore, weaker laser and/or antibody penetration with increasing depth of the tissue may impair the visualization of fluorescent signals at the bottom of the brain volume. Establishing an average for the lower nc82 threshold value by comparing across different brains does minimize this loss of fluorescence during analysis, but the ability to account for nc82 intensity differences deep within the brain is, at present, a limitation of the technique.

We also find that the manner in which flies are processed for fixation impacts the baseline-internalized fluorescence of the dye-exclusion assay. Fixing the entire fly before decapitation attenuates dye influx into the brain; in contrast, the removal of the head or proboscis prior to fixation is associated with a marked infiltration of hemolymph-borne dye into the SEZ of the brain (Figure 2). The mechanism by which decapitation or proboscis removal leads to influx into the brain is unclear, but recently, proboscis extension has been linked to waste clearance from the brain [20] raising the possibility that proboscis activity influences efflux from the brain, though the role of the proboscis in brain influx remains to be determined. It is probable that the mechanical impact of detaching the proboscis from the head leads to the detachment of nerves innervating it from the brain [21]. These nerves are ensheathed by a layer of perineurial and subperineurial glial cells that are continuous with those covering the brain, and such mechanical damage may lead to local disruption of the barrier, thereby facilitating the non-specific influx of tracer into the SEZ [22]. Regardless, the reduced baseline fluorescence achieved with whole-body pre-fixation provided a more sensitive methodology in uncovering the biological effects of traumatic injury on barrier integrity (Figure 3).

In summary, we describe an alternative protocol to the dye-exclusion procedure for assessing barrier integrity in adult *Drosophila*. This workflow focuses on intraparenchymal infiltration and provides *Drosophila* researchers with an additional tool for investigating the cellular and molecular mechanisms that underlie CNS barrier function.

## 4. Materials and Methods

### 4.1. Fly Stocks

Flies were raised at 25 °C at 60% relative humidity on Nutri-Fly Bloomington formulation media (Genesee Scientific, San Diego, CA, USA). 10-day-old female *w^1118^* (Bloomington Stock Center, Bloomington, IN, USA, no. 3605) flies were used for all experiments. 

### 4.2. Dye Injections

The 9-day-old flies were anesthetized on ice and affixed to a pad using double-sided tape. A 5 µL Gastight Syringe (Hamilton, Reno, NV, USA; no. 175 RN) was used to inject approximately 100 nL of 25 mg/mL 10 kDa tetramethylrhodamine dextran (TMRD) (Thermo Fisher, Waltham, MA, USA; D1817) into the thorax of the fly, between the first and second leg. Each fly was allowed to recover at 25 °C. Injection consistency was monitored by qualitative assessment of injected dye in the ventral-posterior region of the proboscis.

### 4.3. Traumatic Injury

To inflict whole-body traumatic injury, we used a high-impact trauma (HIT) device [18]. Briefly, 9-day-old, unanesthetized flies, in groups of 10–20 flies, were placed in a spring-loaded vial 2 h after dye injection. To perform a vial strike, the vial was raised to a 90° angle and released, allowing it to contact a polyurethane pad and causing flies to contact and rebound off the vial edges. One bout of traumatic injury was defined as four vial strikes with five minutes of rest after each strike. Flies were transferred to a new vial with food and allowed to recover for ~24 h at 25 °C. 

### 4.4. Fixation, Brain Dissection, and Immunofluorescence

Whole flies were anesthetized on ice and briefly submerged in 70% ethanol, followed by two brief immersions in 1× Phosphate Buffered Saline (PBS). Flies in the head pre-fixation condition were placed in 1× PBS and microdissection spring scissors (Fine Science Tools, Foster City, CA, USA; no. 15000-08) were used to separate the head from the body. Once the head was separated, the proboscis was either removed using no. 5SF forceps (Fine Science Tools no. 11252-00) or left attached to the head and placed in fixative. Flies that underwent whole-body pre-fixation were placed in the fixative without undergoing any dissection procedures. The whole body or head, with or without the proboscis, was submerged in 4% paraformaldehyde (PFA) for 15 min at room temperature, followed by two brief rinses in 1× PBS. Specimens were then placed in 1× PBS on ice, transferred to a Sylgard dish, and brains were dissected in 1× PBS. Dissection procedure for all conditions included removal of tracheal air sacs surrounding the brain. 

After dissection, whole brains were fixed in 4% PFA for 20 min, washed 2 × 20 min in 1× PBS, and 1 × 20 min in 1× PBS with 0.5% TX-100 (PBT) at room temperature. For the region of interest (ROI) analysis, brains were then dehydrated in 40%, 60%, and 80% glycerol (in 1× PBS) for 10 min each, and mounted in Vectashield with DAPI and cover-slipped using a coverslip bridge. For nc82 binary analysis, after fixation and washing, brains were blocked in 5% Normal Goat Serum in PBT for 30 min at room temperature, and incubated in anti-nc82 (DSHB, Iowa City, IA, USA; 1:2000 in blocking solution) for 2 overnights at 4 °C. Brains were then washed for 3 × 20 min in PBT at room temperature and incubated in goat anti-mouse Alexa 488 (Thermo Fisher; 1:500 in blocking solution) for 2 overnights. Brains were then washed 3 × 20 min in PBT, dehydrated in 40%, 60%, and 80% glycerol (in 1× PBS) for 10 min each, and mounted in Vectashield without DAPI and cover-slipped with a coverslip bridge. 

### 4.5. Imaging and Analysis

Brains were imaged using a laser-scanning Nikon Ti2 confocal microscope under the 20× objective. The top and bottom planes of the imaging window were established using the DAPI or nc82 channel with a step size of 2 µm. For the region of interest (ROI) analysis, a maximum projection image was generated and Nikon NIS Elements software was used to automatically detect an ROI that covered the entire brain, using the DAPI channel. When automatic detection failed to provide an accurate representation of the brain area, the ROI was manually drawn. Dextran-positive, non-nervous tissue found within the ROI, such as tracheal air sacs or fat body, was manually excluded from the ROI. The mean intensity of TMRD fluorescence within the ROI was then quantified. 

For binary analysis using Nikon NIS Elements, intensity thresholds were selected for nc82 and TMRD channels. For nc82, threshold values were first defined to create a binary layer that was representative of the parenchyma for three brains in each group. These values were subsequently averaged together to determine the optimal threshold values used for analysis. For TMRD, low threshold values were determined using a non-injected injured brain to exclude background fluorescence intensity, and high threshold values were not altered. Using these thresholds, individual nc82 and TMRD binaries were created across all z-sections for each brain. An “AND” operation was then applied to both binaries to allow for the selection of pixels that were present within the threshold values of both channels. This led to the creation of a third binary layer, referred to as the intersection binary. This layer was then overlaid over the TMRD channel and total dye fluorescence intensity was measured (for the pixels included in the intersection). The total neuropil brain area (in µm^2^) was also quantified from the nc82 binary for each brain. To account for differences in brain volume, normalized fluorescence intensity was obtained by dividing the total TMRD fluorescence intensity by nc82 neuropil brain area (Supplementary Video S1). 

For binary analysis using FIJI/ImageJ, intensity thresholds were similarly selected for the nc82 and TMRD channels. A binary mask containing pixels that fell within these thresholds was subsequently created across the entire stack for both channels. The intersection of the nc82 and TMRD masks was determined using the “AND” operation under the “Image Calculator” function. This led to the creation of an intersection mask that spanned the entire z-stack. At each individual z-slice of the intersection mask, a ROI was created using the “Create Selection” function and this ROI was overlaid on the TMRD channel to measure total fluorescence intensity. As above, the brain area can be quantified by using the nc82 channel, and normalized fluorescence intensity can be obtained by dividing total TMRD fluorescence by the nc82 brain area (Supplementary Video S2).

### 4.6. Statistical Analyses

All statistical analyses were performed in GraphPad Prism 7.03 (Boston, MA, USA). Data were examined for homogeneity of variance using Bartlett’s and Brown–Forsythe tests. If the data failed either or both of these tests (*p* < 0.05), a nonparametric test was used. A Kruskal–Wallis test, followed by Dunn’s multiple comparisons, was conducted to compare fixation conditions, and a Mann–Whitney U test or *t*-test was used to compare injury and no-injury conditions. The statistical analyses utilized are noted in the figure legend, with error bars representing SEM.

## Data Availability

All data files are available from the Mendeley Data database (doi:10.17632/5ncwxj3z46.1, accessed on 25 October 2022).

## Supplementary Materials

The supporting information can be downloaded at: https://www.mdpi.com/article/10.3390/ijms24031886/s1.
