# Supplementary material for: Optimization and Technical Considerations for the Dye-Exclusion Protocol Used to Assess Blood–Brain Barrier Integrity in Adult Drosophila melanogaster
Source: Int J Mol Sci. 2023 Jan 18;24(3):1886. doi: 10.3390/ijms24031886 (PMC9916281; doi:10.3390/ijms24031886)
Supplement: Supplementary file 1 [file ijms-24-01886-s001.zip › ijms-2019585-supplementary.pdf]

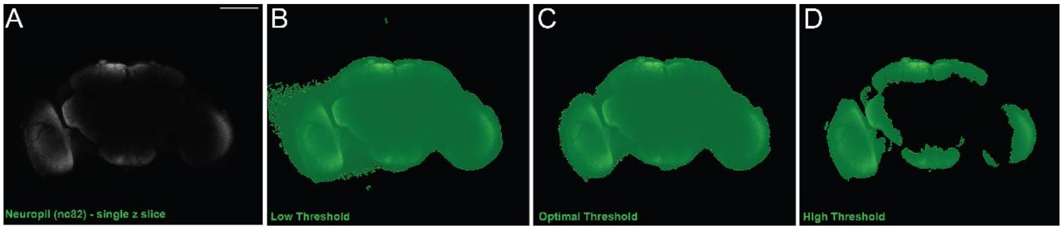

Supplementary Figure S1. Different threshold limits yield varying estimations of parenchymal area. (**A**) Representative laser-scanning confocal z-slice of a d10 w^{1118} female whole-mount brain injected with TMRD on d9 showing neuropil (nc82) staining. (**B-D**) NIS Elements binary analysis of the same z-slice showing: (**B**) overestimation of neuropil area when using a low threshold, (**C**) accurate estimation of neuropil area when using the optimal threshold, and (**D**) underestimation of neuropil area when using a high threshold. Scale bar in: A–D = 100 μm.

Supplementary Video S1. Screen capture video of workflow to assess dye infiltration using NIS Elements software.

Supplementary Video S2. Screen capture video of workflow to assess dye infiltration using FIJI/ImageJ software.
